# Antinociceptive Effect of an Aqueous Extract and Essential Oil from *Baccharis heterophylla*

**DOI:** 10.3390/plants10010116

**Published:** 2021-01-08

**Authors:** Erika Castillejos-Ramírez, Araceli Pérez-Vásquez, Rafael Torres-Colín, Andrés Navarrete, Adolfo Andrade-Cetto, Rachel Mata

**Affiliations:** 1 Departamento de Farmacia, Facultad de Química, Universidad Nacional Autónoma de México, Mexico City 04510, Mexico; erikavivani@gmail.com (E.C.-R.); perezva@unam.mx (A.P.-V.); anavarrt@unam.mx (A.N.); 2 Instituto de Biología, Universidad Nacional Autónoma de México, Mexico City 04510, Mexico; rafael.torres@ib.unam.mx; 3 Laboratorio de Etnofarmacología, Facultad de Ciencias, Universidad Nacional Autónoma de México, Mexico City 04510, Mexico; aac@ciencias.unam.mx

**Keywords:** *B. heterophylla*, Asteraceae, antinociception, infusion, essential oil, quantification, di-caffeoylquinic acids, formalin test

## Abstract

Infusions and poultices prepared from the aerial parts of *Baccharis heterophylla* Kunth (Asteraceae) are widely used in Oaxaca (Mexico) for relieving painful and inflammatory complaints. Therefore, the antinociceptive potential of an aqueous extract (31.6–316 mg/kg, p.o.) and essential oil (30–177 µg/paw, i.pl.) of the plant was assessed using the formalin test. Both preparations inhibited the formalin-induced nociception response (100–316 mg/kg and 100–177 µg/paw, respectively) during the test’s second phase. Chemical analysis of the aqueous extract revealed that the major active components were chlorogenic acid (**1**), 3,4-di-*O*-(*E*)-caffeoylquinic acid (**2**), 3,5-di-*O*-(*E*)-caffeoylquinic acid (**3**), 4,5-di-*O*-(*E*)-caffeoylquinic acid (**4**), 3,5-di-*O*-(*E*)-caffeoylquinic acid methyl ester (**5**), apigenin (**6**), genkwanin (**7**), acacetin (**8**). Compounds **1**–**5** and **8** are new for *B. heterophylla*. A high-pressure liquid chromatographic method for quantifying chlorogenic acid (**1**) and di-caffeoylquinic acids **2**–**4** in the plant was developed and validated. Analyses of the essential oil and the headspace solid-phase microextraction products, via gas-chromatography-mass spectrometry, revealed that the major volatiles were β-pinene, myrcene, D-limonene, β-caryophyllene, and α-caryophyllene, which have demonstrated antinociceptive properties.

## 1. Introduction

As in many rural regions of the world, the population of Capulálpam de Méndez, a Zapotec community with well-preserved nature in the state of Oaxaca (Mexico), strongly depends on traditional herbal medicine to meet their primary health care needs [1]. This town has a Traditional Indigenous Medicine Center, which offers healing services to its inhabitants based on regional plants; this practice is firmly anchored in this community’s social structure and associated with its ancestral culture and history [2]. Thus, the medicinal plants of Capulálpam de Méndez are an essential part of their lives; accordingly, it is critical to analyze them for the development of evidence-based traditional medicines with known quality, security, and efficacy. 

For instituting knowledge-based herbal traditional medicines, whose use is founded on the experience, it is necessary to determine their safety and efficacy using preclinical and clinical assays. When assessing medicinal plants’ efficacy, it is crucial to analyze the traditional remedies and their constituents (e.g., flavonoids, terpenoids, phenylpropanoids, alkaloids, or others), considering that usually the efficacy is accomplished with the mixtures of compounds in these preparations. The mixture of compounds might be acting by synergistic multi-target effects, improving pharmacokinetic parameters or other mechanisms. For quality control of these herbals drugs is important to establish a physicochemical evaluation of crude drug covering aspects, such as selection, macro, and microscopic examination, for detection of the right material, and search of adulterants; qualitative chemical evaluation for identification of single, or a set of constituents (active or not) using chromatography or other analytical procedures; and, finally, quantitative chemical evaluation to estimate the amount of the major classes or constituents, active or not. The processes mentioned involve a wide array of scientific investigations, including physical, chemical, and biological evaluations employing different analytical methods and tools. Altogether, these studies can lead to the development of standardized phytomedicines of good quality [3,4].

One of the medicinal species used in Capulálpam de Méndez for painful and inflammation disorders is *Baccharis heterophylla* Kunth (Asteraceae), a plant widely distributed in Mexico; the aerial parts of this species are consumed in the form of infusions, decoctions, or poultices [5,6,7,8,9,10]. *Baccharis heterophylla* is known as “chamizo de barrer”, “curacuata”, “jarakatua”, and “hierba de la mula”; among others, and it is also employed widely outside Oaxaca for treating painful complaints. Phytochemical investigations of an exudate from *B. heterophylla* recollected in a not specified location of Oaxaca, Mexico, led to the isolation of different types of secondary metabolites, including flavonoids such as apigenin, genkwanin, and naringenin [11], two triterpenes, oleanolic acid, and maniladiol [11,12], a few sesquiterpenoids and two dimeric clerodanes [13]. Previous pharmacological studies have shown that the CHCl_3_−MeOH (1:1) extract of *B. heterophylla* produced a concentration-dependent inhibition of spontaneous ileum contractions and activated Ca^2+^-dependent chloride channels in *Xenopus laevis* oocytes [6,14]. However, the species’ efficacy to treat painful complaints, chemical analysis to determine the major compounds of the traditional preparation, and to establish identity and composition tests remain open questions

Based on the above considerations, the aims of this study were: (i) To investigate the preclinical efficacy of an aqueous extract (AE), and essential oil (EO) prepared from the aerial parts of *B. heterophylla* for treating painful ailments, as well as their chemical composition. (ii) To develop an analytical method using High-Performance Liquid Chromatography (HPLC) to quantify the major components of the traditional preparation. 

## 2. Results and Discussion

### 2.1. Antinociceptive Effect of the Aqueous Extract and Essential Oil

**Figure 1:**
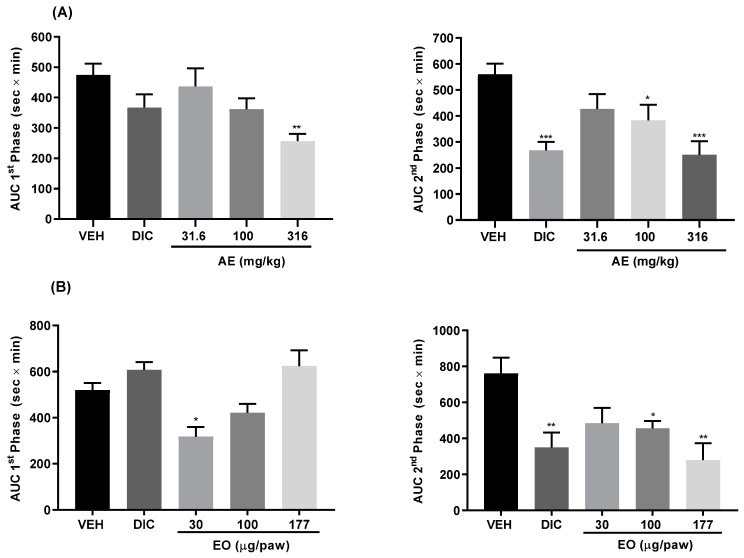
Antinociceptive effect of an aqueous extract (31.6−316 mg/kg) and an essential oil (30−177 µg/paw) from *B. heterophylla* in the formalin test in mice. (**A**) AUC from the time course curve of phase 1 and AUC from the time course curve of phase 2. VEH: vehicle (0.9% saline solution). DIC (50 mg/kg, p.o.). (**B**) AUC from the time course curve of phase 1 and AUC from the time course curve of phase 2. VEH: vehicle (0.9% saline solution). DIC: (100 µg/paw, i.pl.). Each measurement represents the mean ± SEM 6 mice per group. Significantly different from VEH group (* *p* < 0.05, ** *p* < 0.01, *** *p* < 0.001) determined by ANOVA followed by Dunnett’s post hoc test.

The use of infusions and poultices of *B. heterophylla* for treating painful complaints led us to evaluate the antinociceptive activity of an AE and EO obtained from the plant’s aerial parts using the formalin test. This test is regarded as a satisfactory laboratory model for the study of nociception as it encompasses inflammatory, neurogenic, and central mechanisms of nociception. The formalin test is also a useful method for assessing the antinociceptive drugs and elucidating their action mechanisms [15]. The first phase reflects centrally mediated pain with the release of substance P and bradykinin, while the second is due to release of histamine, serotonin, nitric oxide, bradykinin, and prostaglandins [15]. Injection of 2% formalin solution subcutaneously in the hind paw of mice pretreated with vehicle resulted in intense spontaneous licking of the injected paw with a classic biphasic response (Figures S1 and S2, Supplementary Materials). However, oral administration of the infusion (31.6–316 mg/kg) provoked antinociceptive effects in both the first and second phases of formalin-induced nociception; in the first stage (Figure 1A), the effect was only observed at the highest dose (316 mg/kg); the highest responses in the second phase was observed at the doses of 100 and 316 mg/kg, which reduced the licking time significantly. The results were comparable to the action produced by diclofenac (DIC, 50 mg/kg), employed as a positive control drug. On the other hand, intraplantar pretreatment of mice with EO (30–177 µg/paw) also decreased the nociceptive response in both phases of the formalin test (Figure 1B); in the first stage, the highest effect was observed at the concentration of 30 µg/paw, but in the second phase the antinociceptive action was more pronounced at the concentrations of 100 and 177 µg/paw, the results were comparable to the effect produced by positive control (DIC, 100 µg/paw). The overall outcomes suggested that the aqueous extract and essential oil of *B. heterophylla* exhibited antinociceptive action in both phases of the formalin test.

### 2.2. Chemical Constituents of the Aqueous Extract

**Figure 2:**
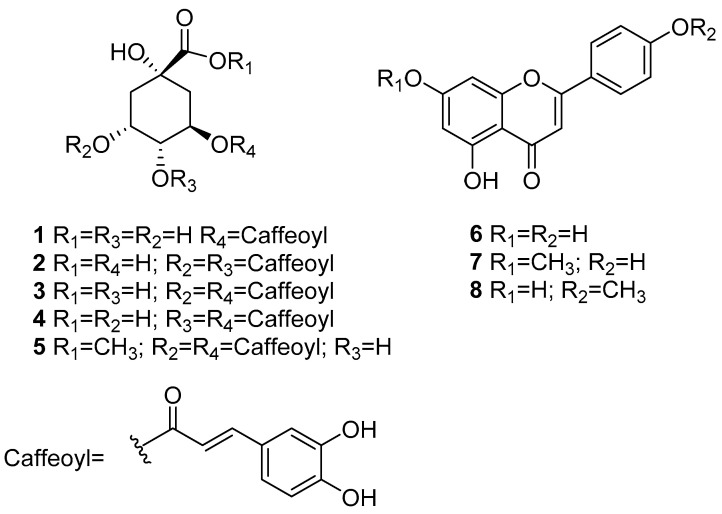
Compounds isolated from *B. heterophylla*’s aqueous extract.

Chemical investigation of the aqueous extract afforded eight known compounds (Figure 2), identified as chlorogenic acid (**1**) [16], 3,4-di-*O*-(*E*)-caffeoylquinic acid (**2**) [17], 3,5-di-*O*-(*E*)-caffeoylquinic acid (**3**) [16,17,18], 4,5-di-*O*-(*E*)-caffeoylquinic acid (**4**) [16,17,18], 3,5-di-*O*-(*E*)-caffeoylquinic acid methyl ester (**5**) [17], apigenin (**6**) [19], genkwanin (**7**) [17], and acacetin (**8**) [20]. The structures of the known compounds were identified by comparing their spectroscopic and spectrometric data with those previously reported or by comparison with authentic samples (Tables S1 and S2, Supplementary Materials). Compounds **1**–**5** and **8** are reported for the first time in this species. 

As with other plant’s traditional preparations [21,22,23,24,25,26,27,28,29], the antinociceptive effects of AE could be related to the high content of chlorogenic acid (**1**), di-*O*-(*E*)-caffeoylquinic acids (**2**–**4**), apigenin (**6**) and acacetin (**8**). Thus, in vivo studies demonstrated that compounds 3,5-di-*O*-(*E*)-caffeoylquinic acid (**3**) and 4,5-di-*O*-(*E*)-caffeoylquinic acid (**4**) showed antinociceptive action in the acetic acid-induced writhing model and the hot plate assay in mouse [21,22]. Compounds **1**, **3**, and **4** significantly inhibited carrageenan-induced rat paw edema [23,24]. In vitro, compound **4** inhibited hypoxia-induced cyclooxygenase-2 (COX- 2), expression and cell migration via a TRPV1-mediated pathway [25]; pretreatment of RAW264.7 macrophage cells with a mixture of di-caffeoyl quinic acids or pure **3** suppressed the production of NO, PGE2, and pro-inflammatory cytokines (TNF-α, IL-1β, and IL-6) by inhibiting the NF-κB and MAPKs pathways ([26,27,28] inter alia). On the other hand, flavonoids **6** and **8** showed antinociceptive effects in different models [29].

### 2.3. Volatile Composition Analyses

The volatile components profile of *B. heterophylla* was gathered by gas chromatography-mass spectrometry (GC-MS) analyses of EO obtained by hydrodistillation and head space-solid phase microextraction (HS–SPME)-adsorbed compounds. The HS-SPME is a more efficient, quicker, and free-solvent method for analyzing volatile constituents from plants and other matrices [30].

**Figure 3:**
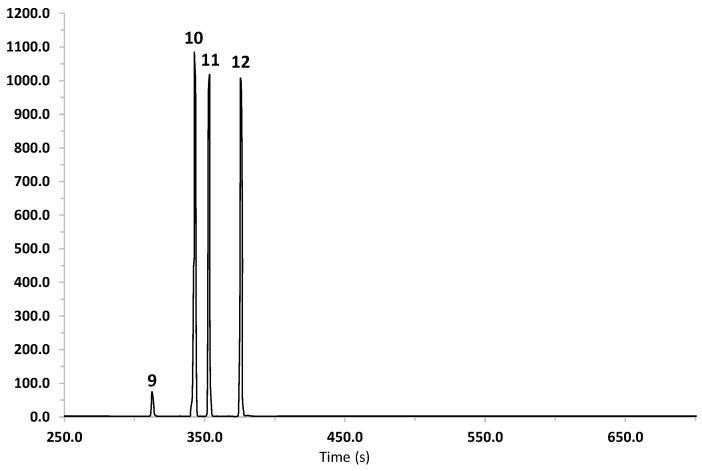
Total ion current chromatogram of the components of the essential oil from *B. heterophylla* extracted using hydrodistillation. Peaks are: α-pinene (**9**), β-pinene (**10**), myrcene (**11**) and D-limonene (**12**).

The results of the study of EO are summarized in Table 1 and Figure 3. The EO of *B. heterophylla* is characterized by a high concentration of monoterpenes, such as β-pinene (28.86%), myrcene (29.57%), and D-limonene (36.24%). These results agree with the chemical composition of essential oils reported for other species of the genus *Baccharis* [31].

For the HS-SPME analysis, four fibers were employed, carboxen/polydimethylsiloxane (CAR/PDMS), divinylbenzene/carboxen/polydimethylsiloxane (DVB/CAR/PDMS), polydimethylsiloxane/divinylbenzene (PDMS/DVB), and polydimethylsiloxane (PDMS). As summarized in Table 1, the analysis with these fibers allowed the identification of 13, 13, 14, and 14 compounds, respectively; their relative percentages and retention indexes are also indicated in Table 1. β-Pinene, D-limonene, and myrcene were the major components detected with DVB/CAR/PDMS and CAR/PDMS stationary phases; this composition is consistent with the main compounds detected in EO. On the other hand, cedrene and β-caryophyllene were the most abundant compounds detected with PDMS and PDMS/DVB-coat fibers.

Altogether, these results revealed that the mixed polarity DVB/CAR/PDMS fiber was the most suitable to establish the volatile composition of the plant’s aerial parts, which is characterized by a high number of hydrocarbons. The total ionic chromatograms of the volatile compounds obtained using this technique are shown in the supplementary materials (Figures S3 and S10).

The major components of the antinociceptive essential oil of *B. heterophylla* are present in several essences, which showed antinociceptive properties [32,33,34,35,36]. β-Pinene (0.3 mg/kg) exhibited antinociceptive action in the hot-place and tail-flick models in rodents [32]. α-Pinene decreased the LPS-induced production of interleukin-6 (IL-6), tumor necrosis factor-α (TNF-α) and inhibited the expression of inducible nitric oxide synthase (iNOS) and cyclooxygenase-2 (COX-2) [33]. At a dose of 10 mg/kg, myrcene was effective in the formalin and hot-plate tests [34]. Besides, myrcene and limonene inhibited IL-1β-induced nitric oxide production in human chondrocytes [35]. Finally, limonene exerted anti-inflammatory and antinociceptive effects in vivo (particularly in chemical models of nociception in mice) and in vitro assays mainly by modulating the action of cytokines and participating in pathways that are closely linked to the inflammatory response [36]. 

### 2.4. Method Validation

**Figure 4:**
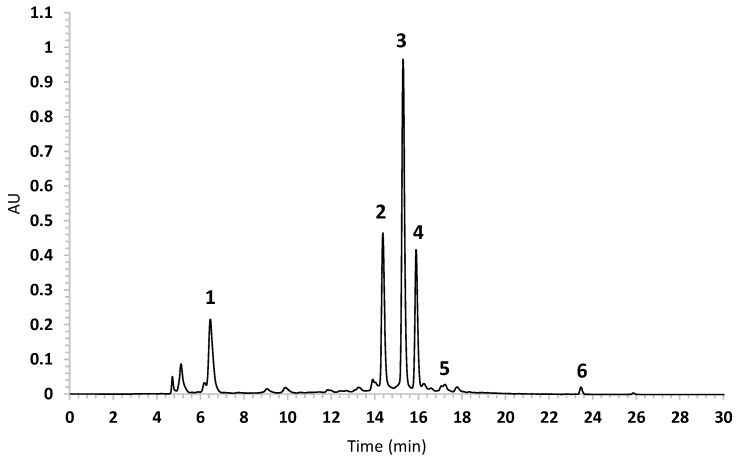
HPLC-PDA chromatogram of *B. heterophylla* aqueous extract under optimized conditions; detection wavelength at 327 nm. Peak identification: **1**: *R_T_* 6 min; **2**: *R_T_* 14.6 min; **3**: *R_T_* 15.6 min; **4**: *R_T_* 16.2 min; **5**: *R_T_* 17.2 min; **6**: *R_T_* 23.6 min.

In the present study, chlorogenic and di-caffeoylquinic acids were selected as markers for the quantitative analysis of *B. heterophylla*. The optimal chromatographic separation was achieved with a Waters HPLC equipped with an XBridge BEH Shield RP18 column and a mixture of water containing 0.1% formic acid and acetonitrile as mobile phase, which was chosen after several trials; the PDA detection wavelength (λ) was set at 327 nm; under these optimized conditions, an effective resolution was achieved for compounds **1**–**4**. Compounds **1**–**4** were identified by their retention time and coelution with AE; a representative chromatogram of the AE of *B. heterophylla* (BH-1) is shown in Figure 4. The developed method was validated using compounds **1**, **3** and **4** according to the International Conference on Harmonization guideline (ICH) [37], and was successfully applied to determine the content of caffeoylquinic acids in AE prepared from different batches of the plant. The overall results are summarized in Table 2 and Table 3.

The method’s selectivity was determined by comparing the chromatographic profile of AE with the data obtained for the standards, considering the retention time and UV spectra (Figures S4–S6, Supplementary Materials). The linearity was tested by analyzing a series of different concentration ranges: 5 to 150 μg/mL for **1**, 20 to 200 μg/mL for **3**, and 13.4 to 133.4 μg/mL for **4**. In all cases, the value of the determination coefficient (*R*^2^) was greater than 0.99. The limits of detection (LOD) and quantification (LOQ) values were 0.5 µg/mL and 1.5 µg/mL for compound **1**, 2.2 µg/mL and 6.6 for compound **3** and 1.4 µg/mL and 4.1 µg/mL for compound **4**. The intraday and interday precision RSDs were no more than 2% while the repeatability variation was no more than 2% (Table 2). Percentage recoveries of the standards are also indicated in Table 2; in each case, a good accuracy was obtained in the range from 99.15% to 100.83% (RSD ≤ 2.0%). Altogether the described previous data revealed that the method was linear, precise and accurate in the range of concentrations evaluated.

The validated method was applied successfully to quantify chlorogenic acid derivatives in four different batches (BH-1, BH-2, BH-3, and BH-4) of the crude drug. The results are presented in Table 3, which shows the content of **1**–**4** was similar in all batches, where 3,5-di-*O*-(*E*)-caffeoylquinic acid was the major component.

## 3. Materials and Methods 

### 3.1. General Experimental Procedures

Melting points were determined on a Fisher-Johns apparatus (Thermo Scientific, Vernon Hills, IL, USA) and are uncorrected. NMR spectra were recorded in a Unity Plus 400 spectrometer (Varian, Palo Alto, CA, USA), at either 400 MHz (^1^H) or 100 (^13^C) MHz, in DMSO-*d*_6_ and MeOH-*d*_4_. Data processing was carried out with the software MestReNova version 12.0.0. Mass spectra of the isolates were obtained on an Acquity UHPLC-H Acquity UHPLC-H^®^Class system (Waters, Milford, MA, USA) equipped with a quaternary pump, sample manager, column oven and photodiode array detector (PDA) interfaced with an SQD2 single mass spectrometer detector with an electrospray ion source. IR spectra were recorded using a Spectrum RXI FTIR (Perkin-Elmer, Waltham, MA, USA). Open column chromatography was carried out on Sephadex LH-20 (GE Healthcare, Urbandale, IA, USA) and silica gel 60, 70-230 mesh (Merck, Darmstadt, Germany).

### 3.2. Reagents

HPLC grade acetonitrile and water, and analytical reagent (AR) grade solvents used for the chromatographic processes were purchased from J.T. Baker (Avantor, Radnor, PA, USA). Chlorogenic acid (1), diclofenac, and Tween 80 were purchased from Sigma-Aldrich (St. Louis, MO, USA). Standards (**3** and **4**) used in the method validation were isolated from *B. heterophylla* (purities ≥ 97%).

### 3.3. Plant Material

*Baccharis heterophylla* was collected in Capulálpam de Méndez, Oaxaca, México on April 2019 (BH-1). The plant material was identified by Rafael Torres Collin and a voucher specimen (1512112) was deposited at the National Herbarium (MEXU), UNAM, Mexico City. In addition, batches from February, July, and October (BH-2, BH-3 and BH-4 respectively) were collected in the same place and the same year, to analyze their content.

### 3.4. Preparation of the Aqueous Extract and Essential Oil

Air-dried aerial parts (leaves and stem) of *B. heterophylla* (5 g) were extracted with 250 mL of boiling water for 30 min. The resulting infusion was filtrated and concentrated in vacuo to yield 1.28 g of dry aqueous extract (EA). On the order hand, the infusion was exhaustively partitioned with EtOAc (250 mL × 3); the combined organic fractions were dried over anhydrous sodium sulfate and concentrated in vacuo to yield 0.116 g of dry ethyl acetate fraction. This process was repeated as much as needed to obtain 10 g of this fraction.

Essential oil (EO) was prepared by hydrodistillation (3 h), with 200 g of fresh aerial parts and 1.3 L of distilled water, using a Clevenger-type instrument. The hydrodistilled mixture was extracted with dichloromethane (250 mL × 3); the combined organic phases were dried over anhydrous sodium sulfate and concentrated in vacuo. This procedure was performed in triplicate and the resulting oils (320 mg) were stored at −4 °C until chemical analysis and pharmacological experiments.

### 3.5. Pharmacological Study

#### 3.5.1. Animals

Male CD1 mice weighting between 30–38 g were obtained from the Bioterium of the School of Sciences, UNAM. Animals were kept on a 12 h light/dark cycle under controlled temperature (22 ± 1 °C) and given a standard pellet diet ad libitum until the beginning of each experiment. Animal experimental protocols followed recommendations of the Mexican Official Norm Animal Care and Handling (NOM-062-ZOO-1999) and were in conformity with international ethical guidelines form care and use of laboratory animals. The experimental protocols were approved by the Institutional Committee for Care and Use of Laboratory Animals (CICUAL-FQ) of Facultad de Química, UNAM (FQ/CICUAL/391/19).

#### 3.5.2. Antinociceptive Effect

The antinociceptive effect of AE and EO was assessed using the formalin test [38]. All the samples were diluted in the vehicle and animals were divided into groups (*n* = 6). Mice were treated with AE (31.6–316 mg/kg, p.o.), EO (30–170 µg/paw, i.pl.), diclofenac (positive control, 50 mg/kg, p.o. and 100 µg/paw, i.pl.) or with vehicle (0.5% Tween 80 in 0.9% saline solution). After 30 min, each animal was received 30 µL of diluted 2% formalin in into the mice dorsal surface of the right hind paw. The biphasic response (licking of the injected paw) induced by the formalin solution was quantified every 5 min during a 30 min period [13]. The doses were logarithmic, and were selected on the basis of previous experimental design for traditional preparations [38]. 

#### 3.5.3. Statistical Analyses

Antinociceptive results are expressed as the mean ± S.E.M. of the analysis of area under the curve (AUC, time of licking against time, sec × min) of mice (*n* = 6), for phases 1 and 2, and both, or as the mean ± S.E.M. of licking time (sec) of mice (*n* = 6) in time courses (Supplementary Materials). Statistical differences were evaluated using either one way ANOVA followed by Dunnett’s in the GraphPad Prism software (version 7; GraphPad Inc., La Jolla, CA, USA). 

### 3.6. Isolation of Compounds

The ethyl acetate fraction (9.5 g) was subjected to column chromatography on Sephadex LH-20, eluting with methanol; 28 (16 mL each) fractions were obtained (F_1_–F_28_). Secondary fraction F_20_ was dissolved in MeOH; from this solution precipitated 85 mg of apigenin (**6**). Column chromatography on Sephadex LH-20 of F_16_ (1.20 g), eluting with acetone−MeOH (9:1), rendered 24 fractions (8 mL each): F_16-1_–F_16-24_. Fractions F_16-5_, F_16-10_, and F_16-13_ yielded 14 mg of **3**, 8 mg of **5** and 5 mg of **4**, respectively. Fraction F_17_ (50 mg) was subjected to column chromatography on silica gel (50 g), eluting with a gradient of hexane−EtOAc (9:1→0:10) and EtOAc−MeOH (10:0→0:10), 12 fractions of 300 mL each were obtained. From F_17-5_, eluted with hexane−EtOAc (7:3), 9 mg of **7** were obtained. From F_17-6_, eluted with hexane−EtOAc (6:4), crystalized 8 mg of **8**. Compounds **1** and **2** were identified by comparison with authentic standards (TLC, HPLC, NMR). 

### 3.7. GC-MS Analyses

All analyses by GC-MS were carried out in an Agilent 6890 N (Agilent Technology, Santa Clara, CA, USA) series gas chromatograph equipped with a LECO Pegasus 4D time-of-flight mass spectrometer detector (IET International Equipment Trading Ltd., Mundelein, IL, USA). A capillary column DB-5 was used [(5%-phenyl)-methylpolysiloxane, 20 m × 0.18 mm I.D.; 0.18 µm film thickness]. The oven temperature was set at 40 °C for three minutes, then at 40 to 300 °C at 20 °C/min and held isothermally at 300 °C for 15 min. Helium was used as the carrier gas at a flow rate of 1 mL/min. Compounds were identified using index retention method (*I_R_*), by co-injection of the sample with a solution containing the homologous series of *n*-alkanes (C_8_−C_27_), and by comparison of their MS fragmentation patterns with those of compounds contained in the spectral database of the National Institute of Standards and Technology (NIST, Gaithersburg, MD, USA). All determinations were performed in triplicate.

### 3.8. Headspace Solid-Phase Microextraction

Volatile compounds of *B. heterophylla* were extracted using the HS-SPME technique; the extraction procedure was conducted as follows: 50 mg of aerial parts, 2 g of sodium chloride, and 15 mL of water (HPLC grade) were mixed in a hermetically sealed vial. Each fiber (CAR/PDMS, DVB/CAR/PDMS, PDMS/DVB and PDMS) was introduced into the vial and exposed to the headspace of the sample for 15 min, at room temperature, keeping the sample magnetically stirred. After sampling, the SPME fibers were directly inserted into the GC injector port and the fibers thermally desorbed. A desorption time of 2 min at 250 °C was used. Before GC–MS analysis, the fibers were conditioned in the injector of the GC system, according to the instructions provided by the manufacturer. All samples were analyzed in triplicate and the relative proportions of individual components adsorbed to the fibers under these conditions was calculated based on the total ion chromatogram (TIC) peak areas as a percentage of the sum of all peak areas. The GC-MS conditions were described in Section 3.7. The SPME fibers used in this study were purchased from Supelco Inc. (Bellefonte, PA, USA). 

### 3.9. HPLC Analyses

All experiments were performed on a Waters HPLC system equipped with a quaternary pump (model 600), photodiode array detector (PDA, model 996), manual injector and an XBridgeTM BEH Shield RP18 column, (130 Å, 5 µm, 4.6 mm × 250 mm, Waters) at a flow rate of 0.8 mL/min. The mobile phase consisted of (A) acetonitrile and (B) water (0.1% formic acid) the following gradient elution program was used: 20–40% A for 0–10 min, 40–100% A for 10–23 min, 100% A for 23–24 min, and 100–20% A for 24–28 min, 20% A for 28–30 min, the injection volume was 20 μL. The UV detector was set at a monitoring wavelength of 327 nm. System control, data collection, and data processing were accomplished using Waters Empower 2 chromatography software.

### 3.10. Method Validation

The HPLC method was validated according to the International Conference on Harmonization Guidelines (ICH, 2005) and included a determination of selectivity, linearity, precision, accuracy, LOD, and LOQ. The linearity of the system was performed through the calibration curves of the standards (**1**, **3**, and **4**); compounds were accurately weighed (5 mg) and dissolved in 5 mL acetonitrile-water (5 mL, 1:5) to prepare stock solutions at a final concentration of 1 mg/mL. The solutions of the calibration curve were obtained by diluting the stock solution in water, ranging from 5−150 μg/mL for **1**, 20−200 μg/mL for **3** and 13.3−133.4 μg/mL for **4**. The linearity was assessed estimating the slope, y-intercept, and the determination coefficient (*R*^2^) using the least-squares analysis. LOD and LOQ were determined based on the standard deviation (σ) of the response and the slope (S) from calibration curve constructed with a series of appropriate concentrations for determination of the limits, using the following equations:

(1) $$LOD=\frac{3.3\;\sigma }{S}$$

(2) $$LOQ=\frac{10\;\sigma }{S}$$

The precision was evaluated using repeatability (intraday) and intermediate precision (interday). Intraday and interday variations were established using six independent replicates of the standard reference of each compound on one and two different days, to determine intraday and interday precision, respectively. In order to study the accuracy of the method, recovery experiments were performed. The samples were spiked with known amounts of the standards, at different concentrations levels (low, medium and high): **1** (5, 50 and 150 μg/mL), **3** (20, 100 and 200 μg/mL) and **4** (13.3, 66.8 and 133.4 μg/mL). The average recoveries were calculated according to the following formula:

(3) $$Recovery\;(\%)\;=\;\frac{amount\;found\;-\;original\;amount}{amount\;spiked}\;\times \;100$$

The relative standard deviation (RSD) was calculated for each determination as a measure of precision and repeatability.

## 4. Conclusions

In summary, the aerial parts of *B. heterophylla* exhibited antinociceptive activity when tested by the formalin assay. The main classes of compounds characterized in the traditional preparation were chlorogenic acid derivatives and flavonoids with known antinociceptive properties. In the case of the essential oil, the activity could be attributed to its high content of antinociceptive monoterpenes. In any case, the pharmacological effects might be achieved via pharmacology synergism and/or polypharmacology. A precise, reliable, and accurate HPLC method for quantifying chlorogenic acid derivatives’ content in the infusion of the plant was developed and validated. This methodology will be useful for preparing standardized infusions or other formulations made up of this species. This method will also be proposed as composition test for the monograph of *B. heterophylla* to be included in the new edition of the Mexican Herbal Pharmacopoeia. Altogether, our results tend to support the medicinal use of *B. heterophylla* to treat painful complaints in Mexican folk medicine and contribute to the rational use of this valuable medicinal plant.

## Figures and Tables

**Table 1:** Volatile constituents from *B. heterophylla* identified by GC-MS obtained by HS-SPME using different fibers and by hydrodistillation.

N°	Compound	*I_R_*		Percent of Each Component			
			EO **	PDMS *	PDMS/DVB *	DVB/CAR/PDMS *	CAR/PDMS *
**9**	α-Pinene	922	5.34	-	-	-	-
**10**	β-Pinene	969	28.86	4.29	4.85	10.6	1.97
**11**	Myrcene	982	29.57	3.43	9.4	27.19	10.53
**12**	*o*-Cymene	1016	-	-	-	-	26.71
**13**	D-Limonene	1019	36.24	4.07	8.82	23.02	26.71
**14**	*p*-Cymenene	1081	-	-	-	-	3.18
**15**	δ-Elemene	1327		2.45	1.34	-	-
**16**	Cedrene	1413	-	19.99	15.62	-	-
**17**	β-Caryophyllene	1414	-	19.99	15.62	9.25	4.23
**18**	Isogermacrene D	1422	-	8.51	5.12	1.89	-
**19**	Aromadendrene	1435	-	3.64	4.01	-	1.92
**20**	α-Caryophyllene	1447	-	7.37	6.95	1.72	5.03
**21**	Germacrene D	1454	-	1.14	1.15	-	-
**22**	γ-Elemene	1470	-	5.6	4.45	8.59	2.7
**23**	α-Selinene	1479	-	1.04	-	-	-
**24**	β-Cadinene	1486	-	-	-	-	4.46
**25**	β-Amorphene	1488	-	-	-	4.77	4.32
**26**	β-Himachalene	1493	-	9.72	8.46	1.27	-
**27**	δ-Amorphene	1510	-	-	3.53	4.3	-
**28**	Calamenene	1511	-	-	-	4.3	3.92
**29**	α-Cadinene	1525	-	-	-	2.45	1.6
**30**	α-Calacorene	1531	-	0.39	0.4	0.64	-
Total (%)			100	91.63	89.72	99.99	97.28

*I_R_*: Index retention on a DB-5 column with reference to *n*-alkanes. * Fibers used for HS-SPME. ** Obtained by hydrodistillation.

**Table 2:** Parameters of validation of method for determination of constituents in *B. heterophylla*.

N°	Linear Range (µg/mL)	Calibration Equation	*R* ^2^	LOQ (µg/mL)	LOD (µg/mL)	Precision		Recovery (% Mean)
						Intraday (% RSD)	Interday (% RSD)	
**1**	5–150	y = 84,372x + 67,400	0.999	1.5	0.5	0.91	1.32	100.83
**3**	20–200	y = 93,446x − 216,607	0.997	6.6	2.2	1.22	1.8	99.15
**4**	13.4–133.4	y = 72,602x − 156,669	0.996	4.1	1.4	1.96	2.0	100.4

**Table 3:** Contents (mg/g) compounds in four batches of the *B. heterophylla* (*n* = 3).

Compound	*R_T_* (min)	Content (mg/g)			
		BH-1	BH-2	BH-3	BH-4
**1**	6	37.9 ± 3.4	39.7 ± 1.7	29.3 ± 1.8	33.9 ± 2.1
**2 ^a^**	14.6	52.4 ± 1.4	61.5 ± 4.5	58.8 ± 3.4	53.1 ± 1.4
**3**	15.6	104.7 ± 3.4	107 ± 12.3	79.8 ± 8.4	99.6 ± 4.4
**4**	16.2	42.1 ± 1.2	44.5 ± 4.3	40.6 ± 4.3	29.3 ± 0.3

^a^ Quantified as **4**.

## Data Availability

Not applicable.

## Supplementary Materials

The following are available online at https://www.mdpi.com/2223-7747/10/1/116/s1, **Figure S1**: Temporal course of the antinociceptive behavior (the licking time against time) and AUC from the time course curve for the Aqueous Extract (AE, 31.6–316mg/kg). Each measurement represented as mean ± SEM of *n* = 6. Significantly different from VEH group (** *p* < 0.01, *** *p* < 0.001) determined by ANOVA followed by Dunnett’s post hoc test, **Figure S2**: Temporal course of the antinociceptive behavior (the licking time against time) and AUC from the time course curve for the Essential Oil (EO, 30–177 μg/paw). Each measurement represented as mean ±SEM of *n* = 6. Significantly different from VEH group (* *p* < 0.05) determined by ANOVA followed by Dunnett’s post hoc test, **Figure S3**: Total ion chromatograms of volatile components from *Bacharris heterophylla* obtained by extraction of HS-SPME, **Figure S4**: Representative HPLC chromatogram at λ = 327 nm, and UV absorption spectrum (200 to 400 nm) of compound **1**, **Figure S5**: Representative HPLC chromatogram at λ = 327 nm, and UV absorption spectrum (200 to 400 nm) of compound **3**, **Figure S6:** Representative HPLC chromatogram at λ = 327 nm, and UV absorption spectrum (200 to 400 nm) of compound **4**, **Figure S7:** Calibration curve (area *versus* concentration) and residual plot for compound **1**, **Figure S8:** Calibration curve (area *versus* concentration) and residual plot for compound **3**, **Figure S9:** Calibration curve (area *versus* concentration) and residual plot for compound **4, Table S1.** ^1^H NMR and ^13^C NMR spectral data of compounds **1**−**5** (400 and 100 MHz respectively; MeOH-*d*_4_) from the aerial parts of *Baccharis heterophylla*, **Table S2.** ^1^H NMR and ^13^C NMR spectral data of compounds **6**−**8** (400 and 100 MHz respectively, DMSO-*d*_6_) from the aerial parts of *Baccharis heterophylla*, **Figure S10**: EI-MS of volatile compounds **9**–**30** from *Baccharis heterophylla.*
