# Supplementary material for: Antinociceptive Effect of an Aqueous Extract and Essential Oil from Baccharis heterophylla
Source: Plants (Basel). 2021 Jan 8;10(1):116. doi: 10.3390/plants10010116 (PMC7827611; doi:10.3390/plants10010116)
Supplement: Supplementary file 1 [file plants-10-00116-s001.pdf]

Antinociceptive effect of an aqueous extract and essential oil from *Baccharis heterophylla*

Erika Castillejos-Ramírez[1], Araceli Pérez-Vásquez[1], Rafael Torres-Colín[2], Andrés Navarrete[1], Adolfo Andrade-Cetto[3] and Rachel Mata [1,*]

[1] Departamento de Farmacia, Facultad de Química, Universidad Nacional Autónoma de México, CDMX 04510, Mexico; erikavivani@gmail.com (E.C.-R.), perezva@unam.mx (A.P.-V.), anavarrt@unam.mx (A.N.)
[2] Instituto de Biología, Universidad Nacional Autónoma de México, CDMX, 04510, Mexico; rafael.torres@ib.unam.mx (R.T.-C.)
[3] Laboratorio de Etnofarmacología, Facultad de Ciencias, Universidad Nacional Autónoma de México, CDMX, 04510, Mexico; aac@ciencias.unam.mx (A.A.-C.)
* Correspondence: rachel@unam.mx (R.M.); Tel.: (+52 55 5622 5289)

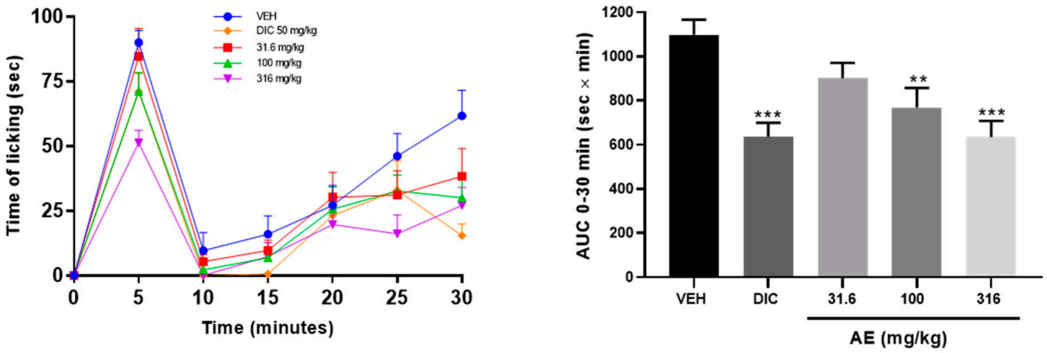

Figure S1. Temporal course of the antinociceptive behavior (the licking time against time) and AUC from the time course curve for the Aqueous Extract (AE, 31.6–316mg/kg). Each measurement represented as mean ± SEM of n=6. Significantly different from VEH group (** $p < 0.01$, *** $p < 0.001$) determined by ANOVA followed by Dunnett's post hoc test.

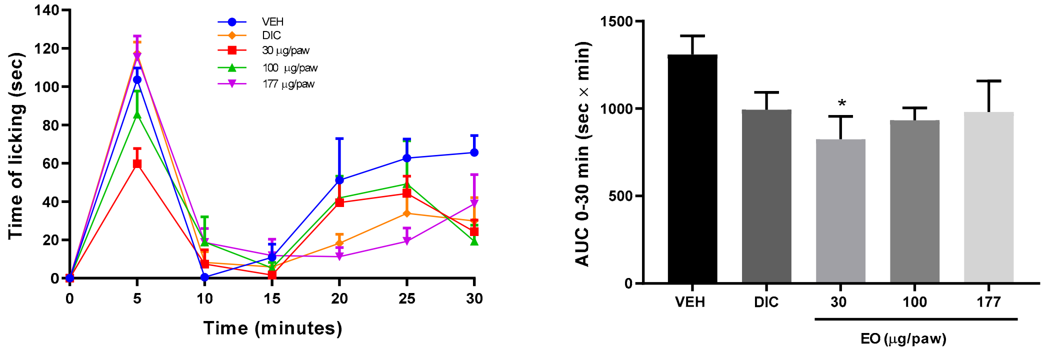

Figure S2. Temporal course of the antinociceptive behavior (the licking time against time) and AUC from the time course curve for the Essential Oil (EO, 30–177 μg/paw). Each measurement represented as mean ± SEM of n=6. Significantly different from VEH group (* $p < 0.05$) determined by ANOVA followed by Dunnett's post hoc test.

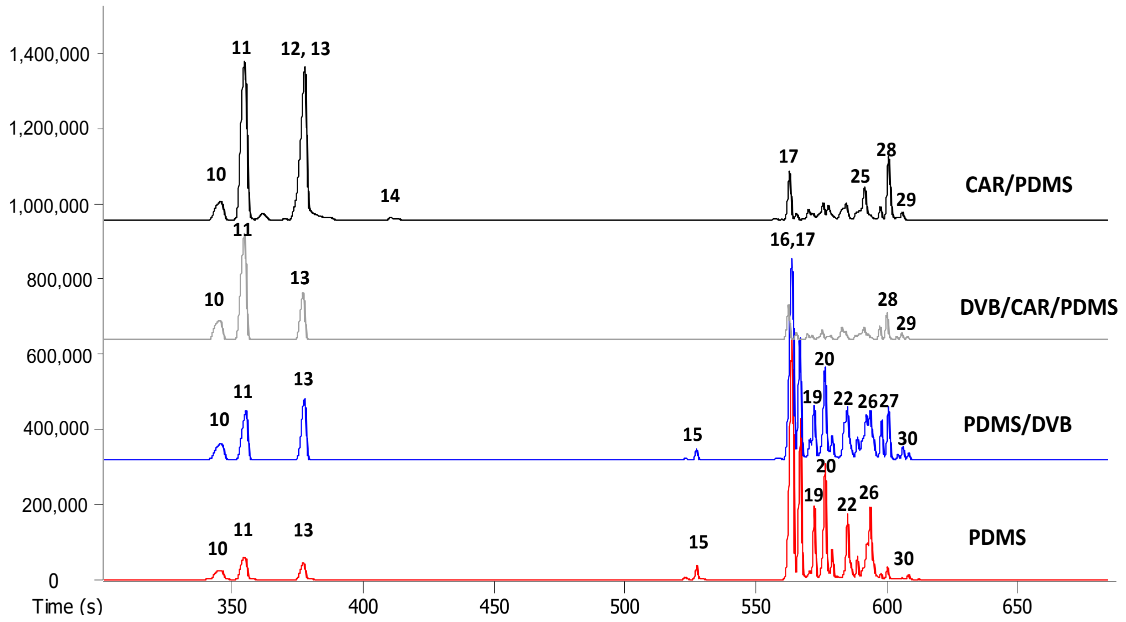

Figure S3. Total ion chromatograms of volatile components from *Baccharis heterophylla* obtained by extraction of HS-SPME.

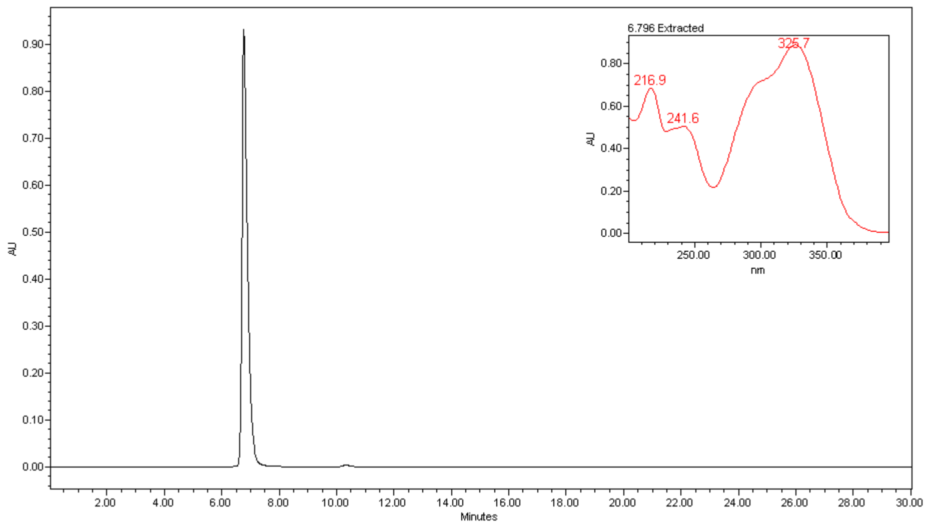

Figure S4. Representative HPLC chromatogram at λ=327 nm, and UV absorption spectrum (200 to 400 nm) of compound **1**.

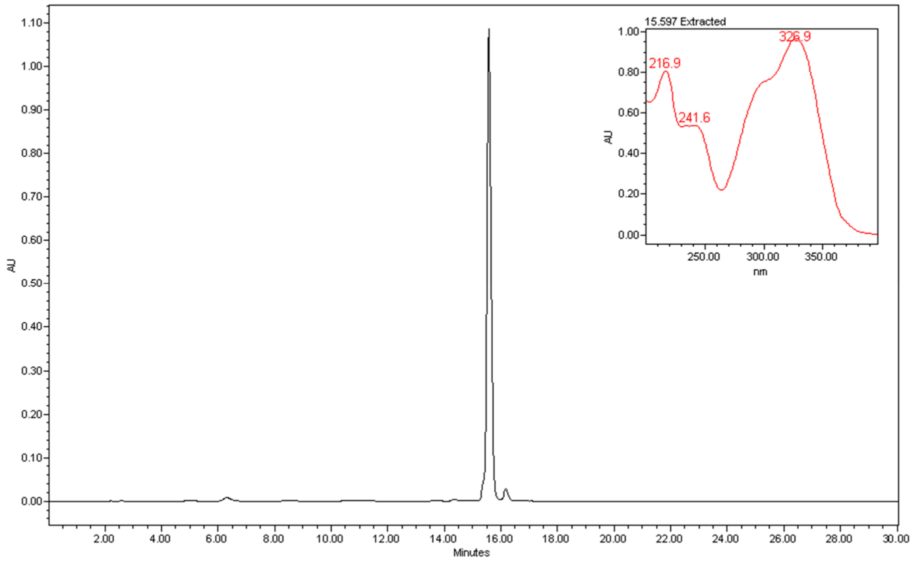

Figure S5. Representative HPLC chromatogram at λ=327 nm, and UV absorption spectrum (200 to 400 nm) of compound **3**.

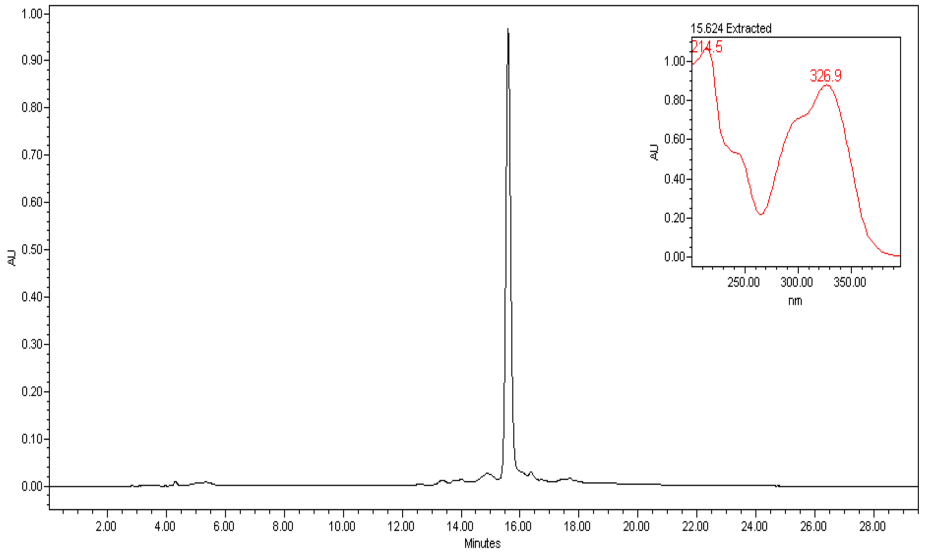

Figure S6. Representative HPLC chromatogram at λ=327 nm, and UV absorption spectrum (200 to 400 nm) of compound **4**.

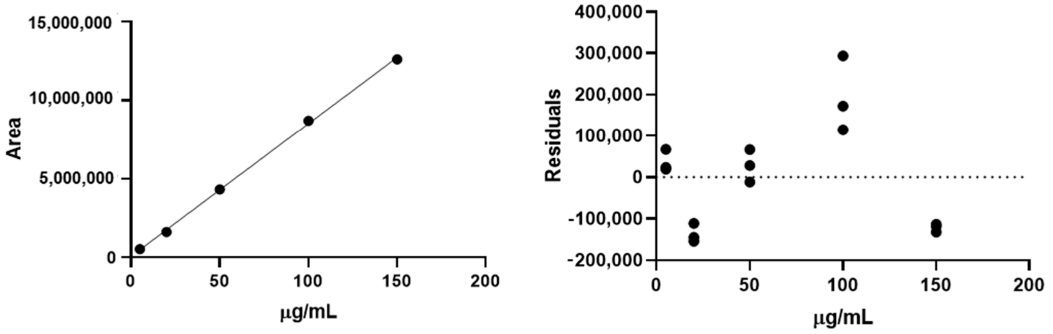

Figure S7. Calibration curve (area *versus* concentration) and residual plot for compound **1.**

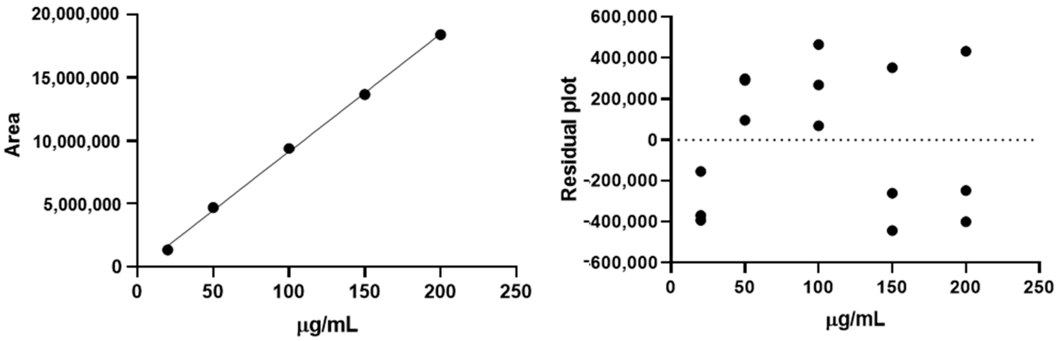

Figure S8. Calibration curve (area *versus* concentration) and residual plot for compound **3.**

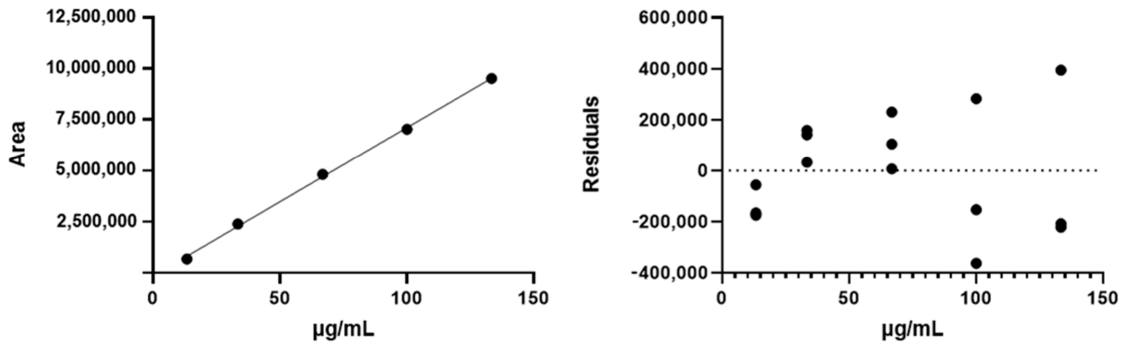

Figure S9. Calibration curve (area *versus* concentration) and residual plot for compound **4.**

Table S1. ^{1}H NMR and ^{13}C NMR spectral data of compounds **1–5** (400 and 100 MHz respectively; MeOH-d_4) from the aerial parts of *Baccharis heterophylla.*

	**1**		**2**		**3**		**4**		**5**	
**Position**	**δ_C**	**δ_H (J in Hz)**	**δ_C**	**δ_H (J in Hz)**	**δ_C**	**δ_H (J in Hz)**	**δ_C**	**δ_H (J in Hz)**	**δ_C**	**δ_H (J in Hz)**
1	74.3		75.1		74.8		80.7		74.6	
2 α/β	36.3	2.20-2.13(m)	37.2	2.05-2.32 (m)	37. 9	2.16-2.37 (m)	38.7	2.09-2.33 (m)	35.5	2.09-2.18 (m)
3	72.1	4.15 (br s)	71.4	5.58(br d, 3.4)	72.7	5.41-5.39 (m)	70.3	4.36 (d, 3.6)	71.9	5.29 (m)
4	74.3	3.70 (dd, 3.2, 9.0)	75.2	5.35(dd, 3.9,9.0)	70.8	3.99 (dd, 3.3, 8.0)	76.7	5.14 (dd, 3.0, 9.7)	69.6	3.96 (m)
5	72.1	5.36 (dd, 3.2, 9.0)	67.0	4.95(dd, 3.3,9.0)	72.1	5.44 (dd, 3.3,8.0)	69.4	5.72-5.67(m)	72.2	5.38 (m)
6α/β	37.5	2.20-2.13 (m)	36.9	2.05-2.32 (m)	36.1	2.16-2.37 (m)	40.3	2.09-2.33 (m)	36.6	2.25-2.34 (m)
7	166.7		177.5		177.7		178.6		175.2	
1′	125.6		127.7		127.9		127.7		127.9	
2′	112.9	7.04 (d, 1.8)	115.3	7.08 (d, 1.9)	115.2	7.08 (br d, 2)	114.9	7.04(d, 2.0)	114.8	7.05 (d, 2.0)
3′	144.5		146.6		146.8		146.7		146.9	
4′	147.3		149.8		149.6		149.6		149.6	
5′	114.2	6.77 (d, 8.2)	116.6	6.80 (d, 8.0)	116.5	6.80 (dd, 0.9, 8.1)	116.4	6.77(d, 8.1)	116.5	6.77 (d, 8.2)
6′	120.7	6.93 (dd, 1.8, 8.2)	123.1	6.92 (dd, 3.0, 8.0)	123.1	6.96 (dd, 2.0, 8.0)	123.2	6.90-6.96 (m)	123.1	6.95 (dd, 2.7, 8.2)
7′	144.7	7.56 (d, 15.9)	147.4	7.59 (d, 15.9)	147.2	7.61 (d, 15.9)	147.6	7.63(d, 15.9)	147.2	7.60 (d, 15.9)
8′	113.2	6.30 (d, 15.9)	115.4	6.25 (d, 15.9)	115.6	6.37 (d, 15.9)	115.0	6.30 (d, 15.9)	115.4	6.32 (d, 15.9)
9′	166.7		168.5		168.9		168.6		167.9	
1″			127.7		127.8		127.6		127.6	
2″			115.3	7.08 (d, 1.9)	115.1	7.08 (br d, 2)	114.9	7.02(d, 2.0)	115.1	7.04 (d, 2.0)
3″			146.6		146.8				146.8	
4″			149.6		149.5		149.6		149.8	
5″			116.6	6.80 (d, 8.0)	116.5	6.80 (dd, 0.9, 8.1)	116.4	6.75 (d, 8.2)	116.6	6.76 (d, 8.2)
6″			12.0	6.92 (dd, 3.0, 8.0)	123.0	6.96 (dd, 2.0, 8.0)	123.1	6.90-6.96 (m)	123.0	6.95 (dd, 2.7, 8.2)
7″			147.4	7.59 (d, 15.9)	147.0	7.61 (d, 15.9)	147.4	7.55(d, 15.9)	147.5	7.53 (d, 15.9)
8″			115.4	6.25 (d, 15.9)	115.6	6.29 (d, 15.9)	115.0	6.22 (d, 15.9)	115.1	6.20 (d, 15.9)
9″			168.5		168.4		168.4		168.8	
OCH_3									53.0	3.67 (s)

Table 2. ^{1}H NMR and ^{13}C NMR spectral data of compounds **6–8** (400 MHz, and 100 MHz respectively; DMSO-d_6) from the aerial parts of *Baccharis heterophylla.*

	**6**		**7**		**8**	
**Position**	**δ_C**	**δ_H (*J* in Hz)**	**δ_C**	**δ_H (*J* in Hz)**	**δ_C**	**δ_H (*J* in Hz)**
2	163.7		167.8		164.0	
3	102.9	6.76 (s)	103.4	6.85 (s)	103.6	6.86 (s)
4	181.7		183.8		182.3	
4a	103.7		105.9		104.4	
5	161.5		157.7		161.4	
6	98.8	6.18 (d, 2.1)	99.1	6.38 (d, 2.1)	99.1	6.19 (d, 2.3)
7	164.1		167.2		163.9	
8	94.0	6.46 (d, 2.1)	93.3	6.78 (d, 2.1)	94.1	6.49 (d, 2.3)
8a	161.2		159.2		157.8	
1′	121.2		118.8		123.3	
2′	128.5	7.91 (d, 8.9)	129.2	7.96 (d, 8.9)	127.9	8.04 (d, 8.8)
3′	116.0	6.91 (d, 8.9)	116.3	6.93 (d, 8.9)	114.3	7.11 (d, 8.8)
4′	157.3		161.8		162.5	
5′	116.0	6.91 (d, 8.9)	116.3	6.93 (d, 8.9)	114.3	7.11 (d, 8.8)
6′	128.5	7.91 (d, 8.9)	129.2	7.96 (d, 8.9)	127.9	8.04 (d, 8.8)
OH-5		12.95 (s)		12.97 (s)		12.91 (s)
OCH3			55.8	3.87 (s)	55.3	3.86 (s)

α-Pinene (9):

Peak True - sample "RM20BA2:4", peak 1, at 312.681 s

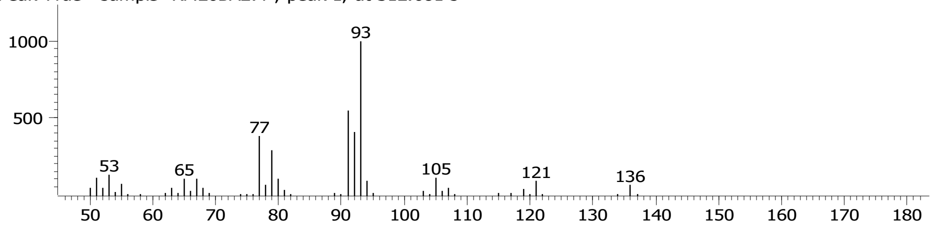

β-Pinene (10):

Peak True - sample "RM20BA2:4", peak 2, at 342.881 s

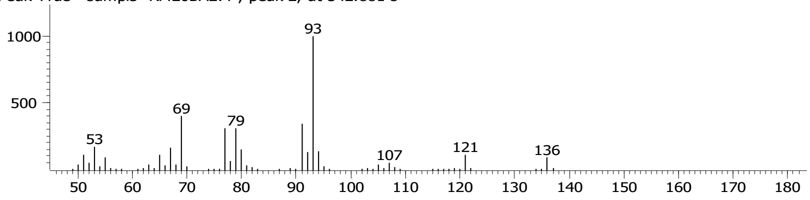

Myrcene (11):

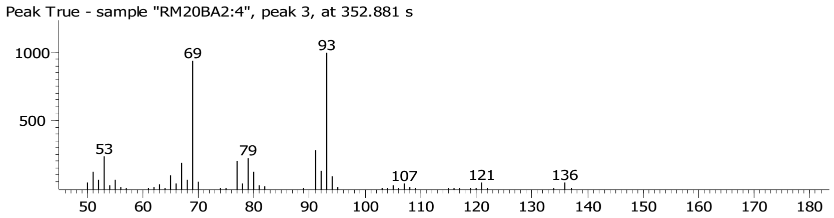

o-Cymene (12):

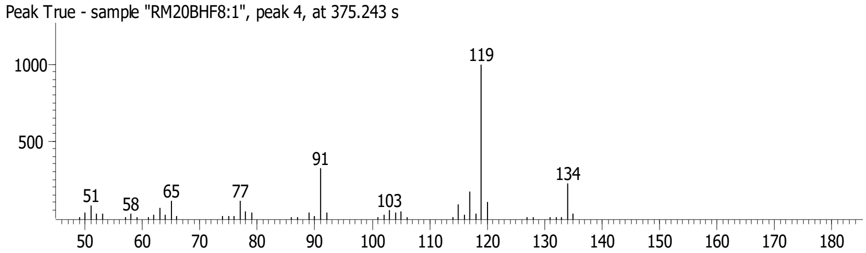

d-Limonene (13):

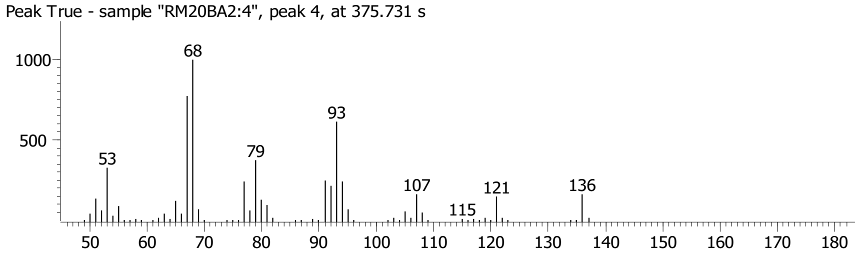

p-Cymenene (14):

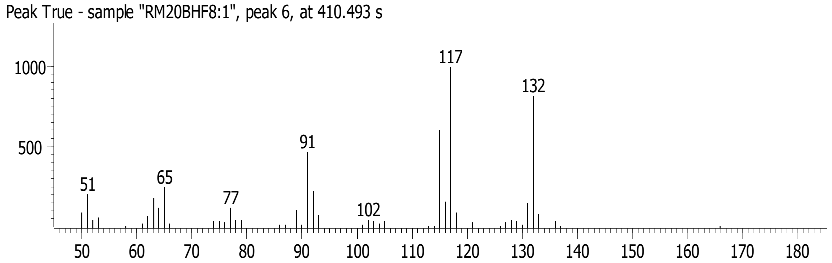

δ-Elemene (15):

Peak True - sample "RM20BHF5:1", peak 4, at 523.194 s

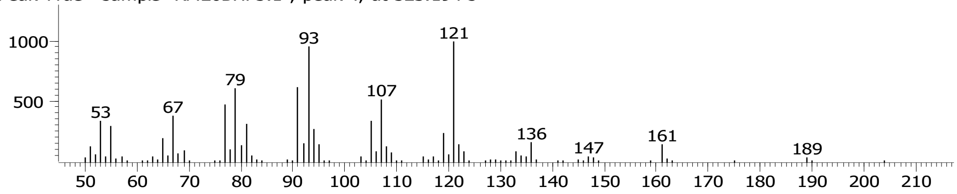

Cedrene (16):

Peak True - sample "RM20BHF5:1", peak 6, at 563.744 s

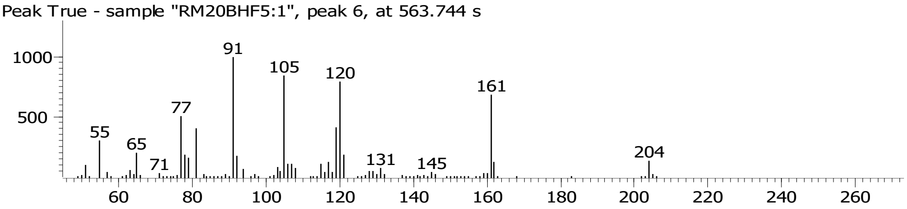

β-Caryophyllene (17):

Peak True - sample "RM20BHF5:1", peak 7, at 564.044 s

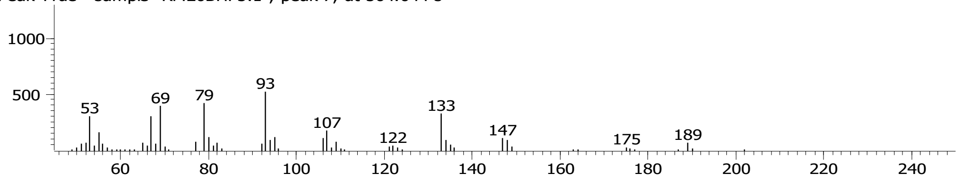

Isogermacrene D (18):

Peak True - sample "RM20BHF5:1", peak 8, at 567.144 s

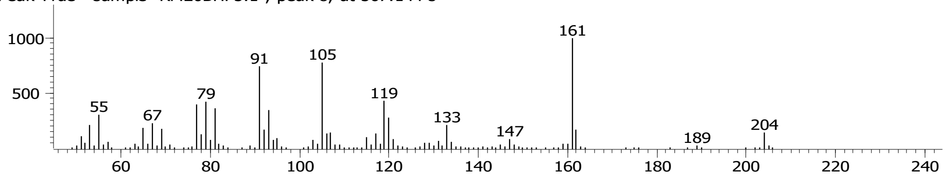

Aromadendrene (19):

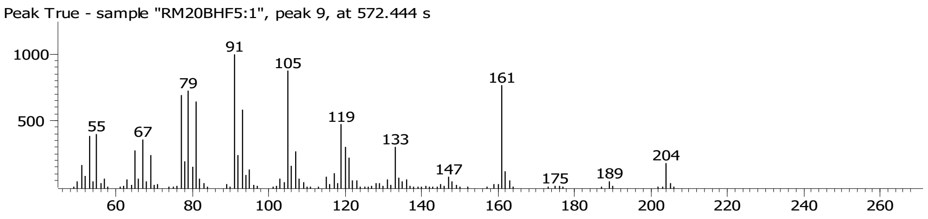

α-Caryophyllene (20):

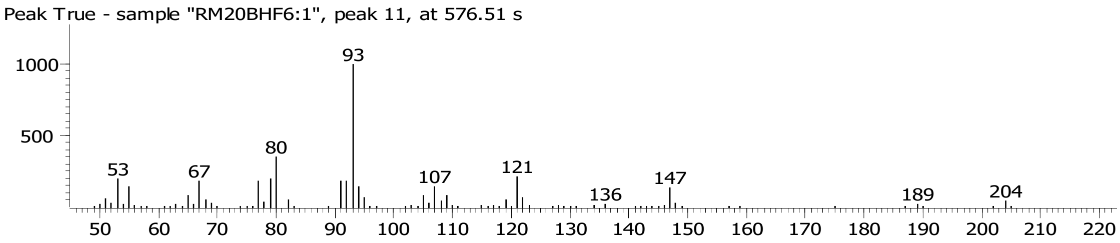

Germacrene D (21):

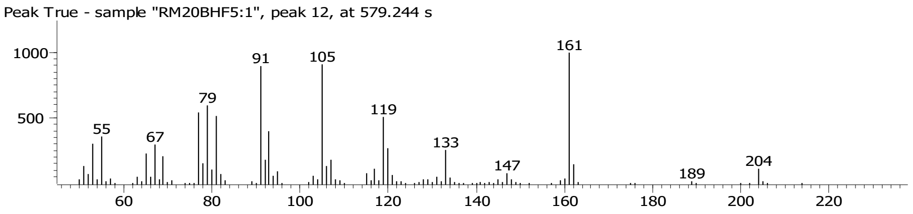

γ-Elemene (22):

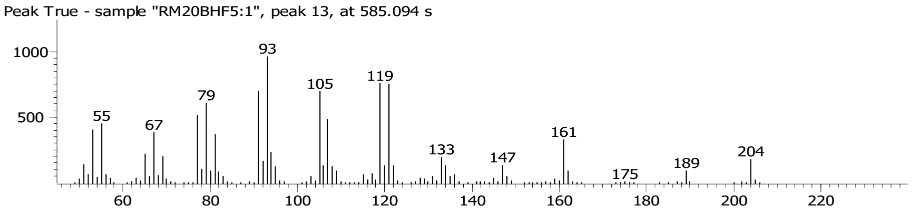

α-Selinene (23):

Peak True - sample "RM20BHF5:1", peak 14, at 588.794 s

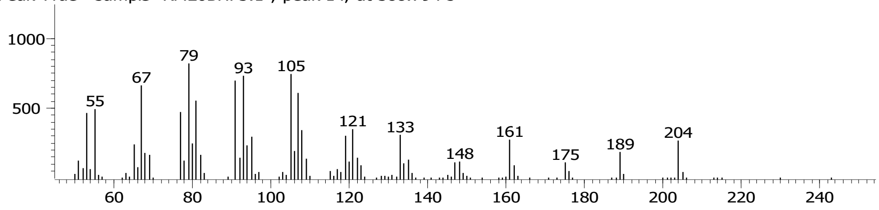

β-Cadinene (24):

Peak True - sample "RM20BHF8:1", peak 12, at 591.443 s

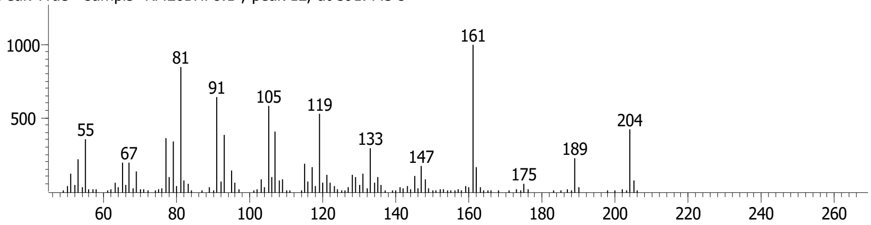

β-Amorphene (25):

Peak True - sample "RM20BHF9:1", peak 14, at 591.325 s

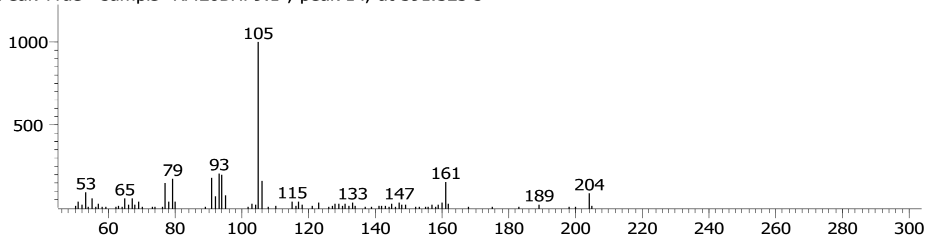

β-Himachalene (26):

Peak True - sample "RM20BHF5:1", peak 15, at 593.794 s

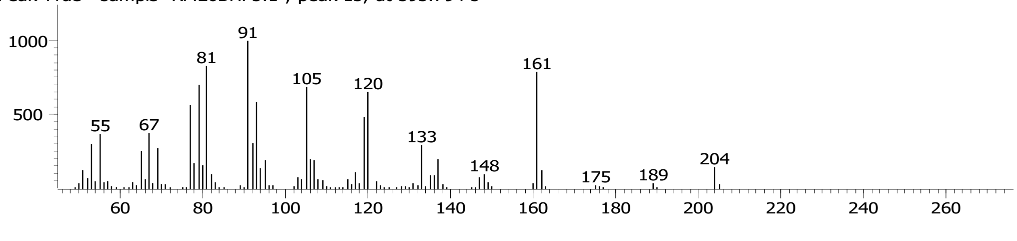

δ-Amorphene (27):

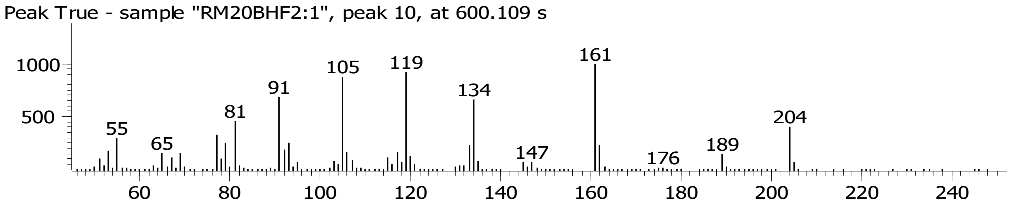

Calamenene (28):

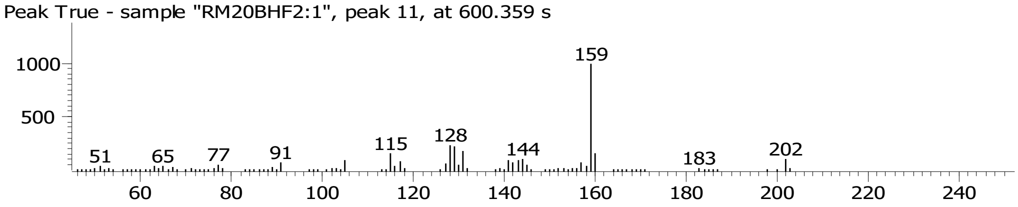

α-Cadinene (29):

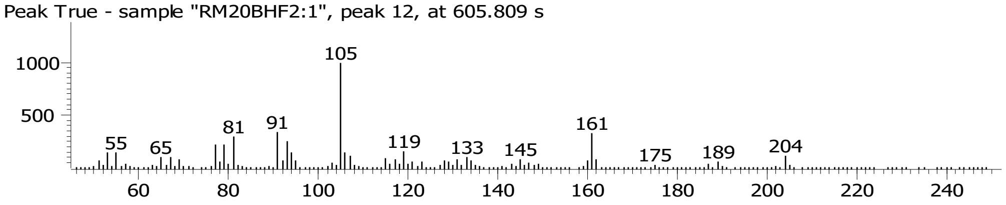

α-Calacorene (30):

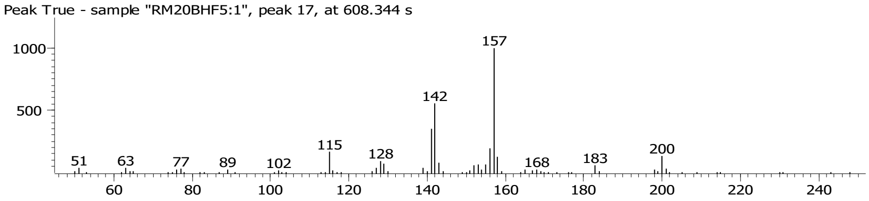

Figure S10: EI-MS of the volatile constituents **9–30** from *Baccharis heterophylla*.
